# Distinctive convergence in Australian floral colours seen through the eyes of Australian birds

**DOI:** 10.1098/rspb.2013.2862

**Published:** 2014-04-22

**Authors:** Martin Burd, C. Tristan Stayton, Mani Shrestha, Adrian G. Dyer

**Affiliations:** 1 National Evolutionary Synthesis Center, Durham, NC 27705, USA; 2 School of Biological Sciences, Monash University, Melbourne, Victoria 3800, Australia; 3 Faculty of Information Technology, Monash University, Melbourne, Victoria 3800, Australia; 4 Department of Physiology, Monash University, Melbourne, Victoria 3800, Australia; 5 Department of Biology, Bucknell University, Lewisburg, PA 17837, USA; 6 School of Media and Communication, RMIT University, Melbourne, Victoria, Australia

**Keywords:** colour vision, convergent evolution, pollination syndrome

## Abstract

We used a colour-space model of avian vision to assess whether a distinctive bird pollination syndrome exists for floral colour among Australian angiosperms. We also used a novel phylogenetically based method to assess whether such a syndrome represents a significant degree of convergent evolution. About half of the 80 species in our sample that attract nectarivorous birds had floral colours in a small, isolated region of colour space characterized by an emphasis on long-wavelength reflection. The distinctiveness of this ‘red arm’ region was much greater when colours were modelled for violet-sensitive (VS) avian vision than for the ultraviolet-sensitive visual system. Honeyeaters (Meliphagidae) are the dominant avian nectarivores in Australia and have VS vision. Ancestral state reconstructions suggest that 31 lineages evolved into the red arm region, whereas simulations indicate that an average of five or six lineages and a maximum of 22 are likely to have entered in the absence of selection. Thus, significant evolutionary convergence on a distinctive floral colour syndrome for bird pollination has occurred in Australia, although only a subset of bird-pollinated taxa belongs to this syndrome. The visual system of honeyeaters has been the apparent driver of this convergence.

## 1. Introduction

Selection exerted by pollinators on floral traits is one of the best studied examples of natural selection arising from biotic interactions [1]. Pollinator-mediated selection can promote the evolution of floral phenotypes that increase the frequency and effectiveness of visits by particular functional groups of pollinating agents [2–4]. Indeed, the morphologies and capacities of particular pollinators are often thought to favour the evolution of convergent suites of floral traits among phylogenetically distant lineages, so-called pollination syndromes [1,5]. Convergence is one of the most powerful demonstrations of adaptation [6], but there has been little quantitative description of the traits composing pollination syndromes, and thus little rigorous testing of convergence.

Convergence for any trait has generally been documented qualitatively, sometimes subjectively, and if qualitative data were used, then no tests for the significance of the pattern have been performed [7,8]. More recently, methods have been developed to quantify the magnitude and significance of observed patterns of convergent evolution [9–11]. Although these methods vary, all require that putatively convergent taxa either be more similar, or have evolved to be more similar, than would be expected by chance [12]. The significance of such similarity is most often assessed via phylogenetic ANOVA [9] or other variance-based methods [10].

For some purposes, however, it is not the degree of phenotypic similarity among putatively convergent taxa that is primarily important; convergence is better identified by the number of times that a given phenotypic state has evolved independently. Such numbers have been documented [7,8], but tests of significance are only now emerging. One new method, SURFACE [11], proceeds by fitting a series of Ornstein–Uhlenbeck models to phenotypic data in a group under study, and determining whether any of the reconstructed adaptive ‘peaks’ are shared among evolving lineages. Although useful for some data, this method does require that lineages evolve in response to adaptive peaks that can be located at single points in phenotypic space. More complicated landscapes, with adaptive ridges or plateaus, for example, cannot, at present, be accommodated with this method. Given that our data suggest this kind of complex landscape, we develop a new method to assess the significance of the convergence observed among bird-pollinated flowers.

Evolutionary shifts to pollination by birds, usually from an ancestral condition of insect pollination, have occurred frequently in a variety of angiosperm lineages [2,13–15]. The novel pollination regime is often—but not always [14]—accompanied by the evolution of novel floral colours [16,17]. Bird-pollinated flowers often have spectral characteristics that correspond to the sensitivities of avian vision [18]. Their reflectance spectra emphasize long-wavelengths, and so appear red or orange to human vision, the conventional floral colours in the bird-pollination syndrome [19,20].

Here, we consider whether the colours of bird-pollinated flowers show evidence of convergent evolution, based on the floral reflectance spectra of 234 native Australian angiosperms, including species visited by birds (80 spp.) and, for comparison, insect-pollinated species (154 spp.). The most important avian floral visitors in Australia are nectarivorous honeyeaters (Meliphagidae) [21], an early diverging family of passerines that diversified in the Eocene [22]. For example, honeyeaters accounted for more than 80% of over 3000 floral visits by birds in the Mount Lofty Ranges and Murray Valley of South Australia [23]. Other pollinating birds in Australia are lorikeets (Psittaculidae), silvereyes and wood swallows (Passerida) [24]. Phylogenetic evidence points to concurrent diversifications of honeyeaters and the bird-pollinated subtribe Embothriinae of the Proteaceae in Australia [25], an indication of the importance of avian pollinator-mediated selection on the Australian flora.

## 2. Material and methods

### (a) Species and floral reflectance

For our analysis, we used the floral reflectance spectra of 211 native Australian angiosperm species from 40 APG III (2009) families gathered by Shrestha *et al*. [18], supplemented with data from an additional 23 native species visited by nectarivorous birds that occurred in the Royal Botanical Gardens Cranbourne, a semi-rural area on the outskirts of Melbourne. Reflectance spectra were measured over wavelengths from 300 to 700 nm on at least two flowers of each species with a calibrated spectrophotometer. Details of the procedure are given in reference [18]. We used the processing functions of the R package *pavo* [26,27] to average multiple spectra within a species, smooth the curves and remove negative reflectance values, which can be introduced by electrical noise in the spectrophotometer.

Each species was placed in one of three floral visitation classes based on data in the literature or on field observations: insect pollination (155 spp.), bird pollination (57 spp.) or visitation by both insects and birds (22 species; electronic supplementary material, table S1). Several species in the latter group come from genera that are usually considered to be bird-pollinated, such as *Epacris* (Ericaceae), *Callistemon* (Myrtaceae) and *Grevillea* (Proteaceae) [24], but if insect visitation has also been reported for a species, then we include it in this group. Nonetheless, we expect that birds have been an important selective agent on floral colour in this group. Thus, we analyse species that are exclusively bird-pollinated together with those that are visited by birds and insects in a collective group that we refer to as ‘bird-visited’ species.

### (b) Phylogenetic tree

In the absence of sequence data for all species in our sample, we assembled a phylogenetic tree from existing phylogenetic analyses. A family-level phylogeny of angiosperms [28] was used as a scaffold and subfamilial topology was added from additional sources (the electronic supplementary material, table S2), leaving polytomies at the genus level where phylogenetic information was lacking. Dating of nodes on the tree was based on the maximum-likelihood dates of reference [29]. A Nexus file of the tree is provided in the electronic supplementary material.

### (c) Colour-space model

We modelled a flower's reflectance spectrum as a single point (colour locus) in a tetrahedral colour space, a representation of the stimulation of the four photoreceptor classes that contribute to colour perception in an avian visual system [30,31]. The spectral sensitivities of avian colour vision fall into two broad classes known as violet-sensitive (VS) and ultraviolet-sensitive (UVS) [32,33]. Honeyeaters, the dominant avian pollinators in Australia, have VS vision [34]. We modelled both types for our analysis. In the tetrahedral colour model, a quantum catch *Q_i_* for each photoreceptor type *i* is calculated based on the photoreceptor sensitivities *R_i_*(*λ*) to wavelength *λ*, the reflectance spectrum *S*(*λ*) of a coloured surface, the spectral distribution of incident light *I*(*λ*) and the background reflectance S_b_(*λ*) to which the photoreceptors are assumed to have chromatically adapted (von Kries adaptation):2.1a
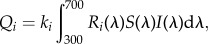
and2.1b
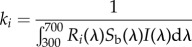
[35,36]. The relative quantum catch values of the four receptor types (normalized to sum to unity) are used to locate a point within a tetrahedron. Each vertex of the tetrahedron represents maximal stimulation of a single photoreceptor type, whereas interior points represent a mix of excitations of the four types. Examples of the use of colour tetrahedrons to interpret avian perception of feather and fruit colours are given by earlier studies [30,31]. Average photoreceptor sensitivities in the VS and UVS types of avian colour vision [37] that we used for our analysis are available in the function *vissyst* of the R package *pavo* [27].

The colour-space locus of floral reflectance was determined for each species in our sample, assuming either VS or UVS receptor sensitivities, incident illumination with a colour temperature of 6500 K (standard noon daylight) [35] and von Kries adaptation to a leaf green background (an average reflectance of living, healthy leaves of nine Australian plant species). Calculations were carried out with the *vismodel* function of *pavo*, and the results were plotted in tetrahedral colour space using the *tcs* and *tcs.plot* functions of *pavo* [27].

### (d) Quantification of convergence and significance tests

We used a phylogenetically informed measure of convergent evolution based on evolutionary trajectories within a tetrahedral colour space. We defined target regions for convergence (such as the space containing all bird-visited species) and used the inferred colour loci of internal nodes in the phylogenetic tree of all sample species to count the number of lineages that evolved from an ancestral state outside the target region to a derived state inside. Potential target regions for convergent evolution were defined by a minimal ellipsoidal hull surrounding the taxa of interest, using the *ellipsoidhull* function of the package *cluster* in R v. 3.0.1 [26] and the Cartesian coordinates defining colour loci of species in the sample (the electronic supplementary material, table S1). Ancestral colour states for all internal nodes of the phylogenetic tree were reconstructed using the *ace* function in the R package *ape* [38]. We then superimposed the colour loci of extant species and the inferred loci of ancestral nodes and extracted the number of branches entering the target region. The greater the number of such evolutionary events, the greater is the degree of convergence. The convergence count included all lineages leading to bird-visited taxa in the target region as well as lineages leading to insect-pollinated taxa that happened to occupy the target ellipsoidal hull. Note that lineage diversification that occurs entirely within a target region does not involve evolution into the morphospace of interest and so does not contribute to the index of convergence. Similarly, subsequent evolution out of the target region does not affect the index.

Because apparent convergence can occur even in the absence of adaptation, especially in low-dimensional morphospaces such as the colour tetrahedrons, we tested the significance of the observed convergence indices by simulating floral colour evolution on the phylogenetic tree according to a Brownian motion model of evolution, with the evolutionary variance–covariance matrix estimated from the observed data. We ran 999 simulations for each target region in each colour space and scored the convergence index in each simulation to establish a null distribution. This null distribution was used for significance tests. All calculations were performed in R v. 3.0.1 using the *convevol* package (written by C. T. Stayton), which uses routines from the *ape*, *cluster*, *geiger* and *phytools* packages [38–41].

## 3. Results

### (a) Colour space

**Figure 1.**
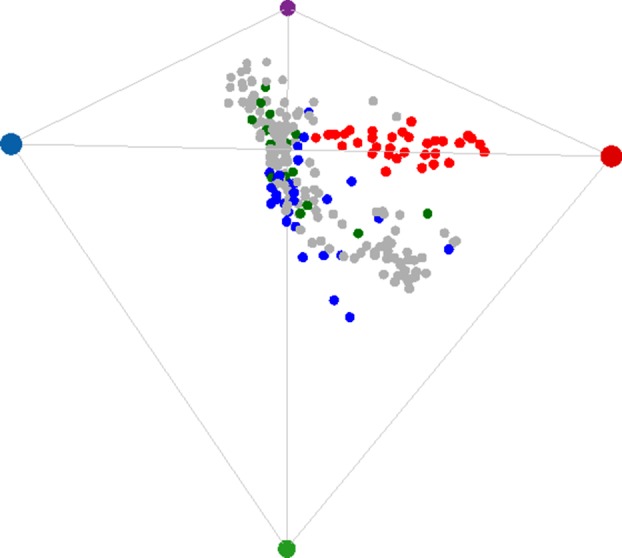
Colour loci in the tetrahedral model for avian VS vision. Vertices represent maximal excitation of the violet, blue, green and red photoreceptors of VS vision. Blue and red vertices are in the foreground; violet and green vertices are in the background. Each point represents the reflectance pattern of flowers of one angiosperm species. Red points: bird-visited species making up the ‘red arm;’ blue points: non-red-arm flowers pollinated by birds; green points: non-red-arm flowers visited by both insects and birds; grey points: flowers pollinated by insects.

**Figure 2.**
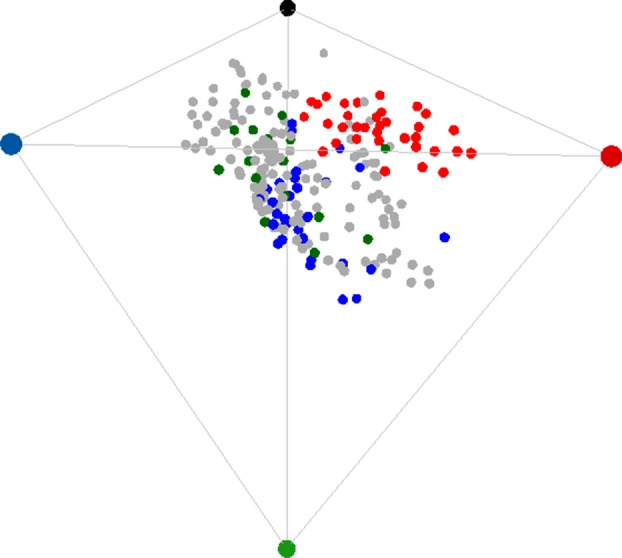
Colour loci in the tetrahedral model for avian UVS vision. Interpretation is the same as in figure 1, except that one vertex of the tetrahedron (black) corresponds to the UV photoreceptor of UVS vision.

Flowers of the 234 Australian species formed distinctive patterns in colour space. The minimum convex hull surrounding all colour loci in the VS colour space occupied 22.3% of the total tetrahedral volume (figure 1), whereas the same species in UVS colour space occupied 43.5% of the total volume (figure 2). These patterns correspond to two features of colour vision. First, natural colours typically occupy only a portion of the total volume of a colour space, because some regions in the space that would require excitation of only one or two receptor types are physiologically unattainable, owing to overlap in the spectral sensitivities of receptors [42], or because of limitations to the hues and saturations that natural pigments can produce [35]. Second, occupation of a larger volume of UVS space than VS space by the same set of floral spectra corresponds to the capacity for finer colour resolution of the UVS visual system, one of the presumed adaptive advantages of this derived trait [43].

**Figure 3.**
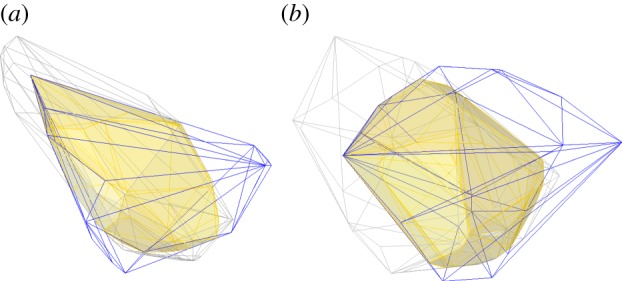
Overlap of floral colours of insect-pollinated species and bird-visited species in tetrahedral colour space. (*a*) VS vision; (*b*) UVS vision. Tetrahedral outlines are not shown for convenience of display, but the red vertex lies to the right. Minimum convex hulls containing all insect-pollinated species are outlined in grey; equivalent hulls for all bird-visited species are outlined in blue. The region of overlap is indicated in yellow shading. In VS space (top), the overlap is 67.3% of the volume occupied by insect-pollinated species and 44.7% of the combined volume encompassing all taxa. In UVS space (bottom), the equivalent values are 64.0% of the volume for insect-pollinated species and 38.1% of the combined volume for all species.

The colour loci of insect-pollinated species occupy a long arc in VS colour space stretching from near the violet apex to the green–red edge of the tetrahedron (yellow flowers to human vision), with a few outlying species (figure 1). The floral colours of bird-visited species are similar to those of insect-pollinated species. The minimum convex hull of VS colour space surrounding all bird-visited species overlapped 67.3% of the volume occupied by insect-pollinated species (figure 3). However, the bird-visited species fell into two relatively distinct groups: 46 species had colour loci that largely coincided with the arc of insect-pollinated taxa, whereas 34 other species fell in a narrow region of the colour space extending from the middle of the insect-pollinated arc towards the red vertex of the tetrahedron (figure 1). This ‘red arm’ occupies only 6.2% of the minimum hull containing all colour loci (1.3% of the total tetrahedral volume) and overwhelmingly contains bird-visited taxa (27 species exclusively bird-pollinated, six visited by birds and insects and one insect-pollinated). Of these 34 species, 20 are recorded as having honeyeaters as visitors; others may be visited by honeyeaters, but the information on pollinators available in the literature does not always provide such precision.

**Figure 4.**
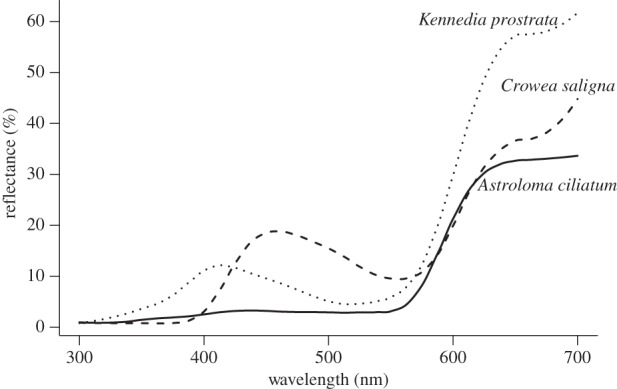
Floral reflectance spectra of three ‘red arm’ species, showing a range of saturation in long wavelengths.

The characteristics of red arm colours can be seen in the reflectance spectra of representative species (figure 4). The colour locus of *Crowea saligna* lies at the base of the red arm near the arc of insect-pollinated species. While its flowers reflect long (red) wavelengths quite strongly, they also have considerable reflectance at shorter wavelengths from about 420 to 520 nm. Thus, these flowers have a relatively unsaturated colour that appears pink or violet-red to human vision. *Kennedia prostrata* has a floral colour locus about midway along the red arm. Like *C. salgina* flowers, *K. prostrata* flowers have reflectance peaks at long and short wavelengths, but both the absolute and relative reflectance at long wavelengths is greater than at short wavelengths, and the red of these flowers is more saturated. *Astroloma ciliatum* lies at the tip of the red arm near the red vertex of the colour tetrahedron. It has little reflectance except at long wavelengths and presents a highly saturated red colour.

Between the red arm species and the remaining taxa lies a gap of unoccupied colour space (figure 1), suggesting that two distinct classes of flower colours exist among bird-visited species. Moreover, congeneric species of bird-visited taxa typically occurred on opposite sides of this gap (the electronic supplementary material, figure S1), a pattern consistent with selection favouring the particular colours that constitute the red arm.

The main features of the distribution of floral colours in UVS colour space are similar to those in VS space, although both the red arm and the gap between it and the remaining colour loci appear less distinct (figure 2). There was only slightly less overlap in floral colours between the two main pollination groups: 64.0% of the minimum convex hull containing all insect-pollinated species overlapped the hull surrounding bird-visited species in UVS colour space (figure 3). The red arm species occupied more than twice the volume of UVS space that they did in VS space, but because all colour loci were more dispersed, the red arm represented 6.9% of the minimal convex hull of all colour loci, similar to the equivalent figure of 6.2% in VS space.

### (b) Convergence

By the criterion that convergent branches of the phylogenetic tree must originate at an ancestral state outside a target region and terminate inside it, there were no convergences on the region of VS colour space encompassing all bird-visited species, and only two convergences on this region in UVS space (table 1). However, when the target region was restricted to bird-visited species in the red arm, the observed number of convergences rose to 31 in both VS space and UVS space (table 1). The red arm target volume is little changed when defined by species pollinated only by birds, or by birds and insects; thus, the convergence index remains high for these targets, with 25 or 26 convergences in VS space and 19 or 28 in UVS space (table 1). Finally, if we define the target by bird-visited species outside the red arm, the reconstructed root state lay within this region and the convergence index was only seven occurrences in VS space and 14 in UVS space (table 1). Outside the red arm, the two subgroups of bird-only and bird-and-insect visitation occupied smaller, somewhat separate regions (figures 1 and 2) that did not necessarily contain the root state. Correspondingly, the number of convergence events was higher for these two subgroups than for the target defined by all bird-visited species (table 1).

**Table 1.** Number of convergence events on target regions of colour space. Target regions are the minimum ellipsoidal hull surrounding the species indicated. *p* indicates the probability of obtaining the observed number of convergent branches in the phylogenetic tree given the null distribution obtained by simulation of Brownian motion evolution of floral colour. Significant probabilities after table-wide sequential Bonferroni correction are indicated in italics.

colour space	target region	observed convergences	Brownian motion		*p*
			mean ± s.d.	max.	
all bird-visited species					
VS	all species in group	0	2.70 ± 2.69	14	1
VS	‘red arm’ species	31	5.49 ± 3.78	22	*0.001*
VS	non-‘red-arm’ species	7	3.52 ± 3.06	19	0.159
UVS	all species in group	2	2.73 ± 2.76	15	0.541
UVS	‘red arm’ species	31	5.70 ± 3.78	22	*0.001*
UVS	non-‘red-arm’ species	14	3.53 ± 3.01	20	0.055
species with only bird pollination					
VS	all species in group	7	4.21 ± 3.32	19	0.665
VS	‘red arm’ species	26	9.22 ± 5.07	26	*0.002*
VS	non-‘red-arm’ species	33	8.28 ± 4.67	26	*0.001*
UVS	all species in group	7	4.18 ± 3.35	20	0.151
UVS	‘red arm’ species	28	9.46 ± 5.11	32	0.008
UVS	non-‘red-arm’ species	36	7.91 ± 469	34	*0.001*
species with bird and insect visitation					
VS	all species in group	13	6.61 ± 4.28	26	0.528
VS	‘red arm’ species	25	6.70 ± 5.00	15	*0.001*
VS	non-‘red-arm’ species	20	8.32 ± 4.74	33	0.023
UVS	all species in group	11	6.48 ± 4.02	21	1
UVS	‘red arm’ species	19	6.73 ± 4.36	16	*0.001*
UVS	non-‘red-arm’ species	15	8.69 ± 4.87	38	0.115

In both VS and UVS colour spaces, the minimum ellipsoidal hull surrounding all bird-visited taxa was large and contained the reconstructed root state for the phylogeny. Thus, a lineage would need to have evolved out of the bird-visited colour space and then return to it in order to contribute to the convergence index. Such events did occur under Brownian motion evolution; indeed, they occurred more often than was observed in the empirical data (table 1). Thus, the convergence indices for this target region were non-significant (*p* = 1 for VS space; *p* = 0.541 for UVS space), and we have no evidence that bird-pollinated flowers, in general, have a floral colour syndrome produced by adaptive convergence.

By contrast, the large observed number of convergences on the red arm region for all bird-visited taxa is very unlikely to have occurred by chance (*p* = 0.001) in either the VS or UVS tetrahedrons, given that no simulation run produced more than 22 convergences in either colour space (table 1). The convergence indices were also significant for the red-arm region of VS space defined by the two subgroups (table 1), but not for the red arm in UVS space containing species pollinated only by birds (table 1).

The bird-visited species lying outside the red arm define a region with non-significant convergence indices (*p* > 0.05) for both VS and UVS colour spaces (table 1). Convergence remains non-significant after sequential Bonferroni correction for the non-red-arm regions defined by bird-and-insect-pollinated species, but significant convergence occurred on the non-red-arm region defined by species with bird pollination only (table 1). Because congeneric species often occur on both sides of the unoccupied gap in colour loci (electronic supplementary material, figure S1), the significant convergence on both the red arm and non-red arm regions, in this instance, may reflect processes that produce divergence in floral colour within lineages.

## 4. Discussion

The reality of pollination syndromes has been contested, because many angiosperm species have generalized relationships with multiple classes of pollinators [1,44], and because floral traits do not seem to cluster into distinct regions of morphospace corresponding to the putative syndromes [45]. These demurring views have some support in our analysis. The colours of Australian flowers visited by birds are not, as a whole, very different from the colours of flowers visited only by insects (figure 3), suggesting that there is no general syndrome for floral colour that is distinct to bird-pollination. Furthermore, lineages with bird-visited flowers have not converged on their total collective region of colour space more often than would be expected by chance. These features of the flowers in our dataset hold true for the colour perception of either VS or UVS avian vision.

However, while bird-visited flowers as a whole may not conform to a discrete and distinct colour syndrome, there appears to be a subset of species that does. Colour loci for these flowers occupy a small volume of the tetrahedral colour space that we have called the red arm, as they form a roughly linear cluster corresponding to strong excitation of the red photoreceptors of avian vision. This cluster contains variation in colour saturation that could readily evolve through modulation of the relative activity of biosynthetic pathways of floral pigments, a mechanism known to produce colour variation [16,17,46,47]—variation in saturation, in particular [48]—in other taxa. Colour differentiation within the red arm could be the product of selection to differentiate floral colour signals among species within the red arm. Much more investigation of current and reconstructed geography and community ecology would be required to establish whether competition for pollinator attention was a likely selective force on flower colour among the taxa involved.

An analysis by Ollerton *et al*. [45] that included morphological, nectar and odour traits in addition to flower colour showed that while descriptions of pollination syndromes clustered in distinct regions of trait-space, actual flowers tended not to form discrete clusters and, more importantly, seldom overlapped the regions occupied by the canonical syndromes. The discrete coding of traits used in the Ollerton *et al*. [45] analysis—in particular, the use of discrete floral colour categories based on human vision—may have inflated the apparent separation of syndromes in trait-space. Our continuous quantitative representation of floral reflectance spectra in a colour-space relevant to floral visitors is more realistic, but we have considered only one key element of traditional pollination syndromes. Nonetheless, repeated convergent evolution of similar floral reflectance patterns among lineages with the same or similar floral visitors is noteworthy and within the conventional thinking about the selective origin of pollination syndromes. While some presentations of the syndrome concept may have overstated the discreteness and ubiquity of syndromes, a nuanced view that does not demand that all species with a particular pollinator conform to a syndrome or to all its parts strikes us as a useful element of our conceptual toolbox.

The loci of the red arm species are much more cohesive and discrete in VS colour space than in UVS colour space (compare figures 1 and 2). Thus, flowers in this group would likely have more distinctive coloration in comparison with flowers outside the group in the eyes of an avian pollinator with VS vision than one with UVS vision. Honeyeaters (Meliphagidae), the most important group of nectarivorous birds in Australia, have VS vision, as they diverged early in the passerine radiation [49] before the evolution of the UVS visual system that characterizes the Passerida [34]. Selection on bird-pollinated Australian angiosperms since the Eocene was likely to have been dominated by this family [25]. Hence, selection on floral colour is likely to have been shaped by the perceptual attributes of VS vision. Nectarivorous passerids with UVS vision exist in Australia [24], and we cannot entirely discount the role of avian UVS vision in pollinator-mediated selection. Nonetheless, the correspondence between the visual characteristics of the dominant avian nectarivores in Australia and the distinctiveness of our red arm species in VS colour space is consistent with the evolution of a colour syndrome for bird pollination in Australia. Only about half the bird-visited species in our dataset would belong to this syndrome, but we see no reason why the term ‘syndrome’ should not be used to describe those species that do fall within such a limited and readily interpretable region of a morphospace.

Avian nectarivores in other regions of world come from evolutionary lineages that are absent or relatively unimportant in Australia. In particular, hummingbirds (Trochilidae) are currently an exclusively New World lineage, whereas sunbirds (Nectariniidae) are principally African and south Asian [13]. Hummingbirds have a VS visual system, whereas sunbirds have the derived UVS system common to passerids [34]. It is currently not known how the visual systems of these floral visitors have affected the evolution of floral colours on other continents, although the VS vision of both honeyeaters and hummingbirds might be expected to produce convergence on the red arm region of colour space in both American and Australian floras. We would expect a red arm among hummingbird-pollinated flowers to be more distinct in the VS space than UVS space, analogous to the pattern in our Australian sample (figures 1 and 2). For example, floral bracts of hummingbird-pollinated *Heliconia* species in the neotropics reflect strongly in long wavelengths [50], similar to the spectra of red-arm species in this study (figure 4). This similarity is consistent with the hypothesis of intercontinental convergence, but much additional data will be needed to fully explore and test this hypothesis. By contrast, the distribution of colour loci of African and south Asian flowers with sunbird visitors may have a more coherent pattern in the UVS space than in the VS space, if similar visual-based selection on floral colour has occurred.

## Data accessibility

Phylogenetic tree: electronic supplementary material, Burdetal_Austr_tree.nex. List of taxa, pollination class, location in or out of the ‘red arm’ region and Cartesian coordinates in VS and UVS colour space for each species: electronic supplementary material, table S1.
